# Pharmacokinetics, Safety, and Exploratory 12-Month Clinical Follow-Up of a Novel BIOtorcin™-Coated Balloon for Femoropopliteal Artery Disease: The BRight PK Study

**DOI:** 10.3390/biomedicines14102359

**Published:** 2026-10-16

**Authors:** Bernardette Jingfei Lee, Davina Daudu, Isabel Dwyer, Daniela Liezel Mwipatayi, Jacqueline Wong, Bibombe Patrice Mwipatayi

**Affiliations:** 1 Department of Vascular and Endovascular Surgery, Sir Charles Gairdner Hospital, Perth, WA 6009, Australia; 2 Department of Vascular and Endovascular Surgery, Royal Perth Hospital, Perth, WA 6000, Australia; 3 Department of Vascular and Endovascular Surgery, Joondalup Health Campus, Joondalup, WA 6027, Australia; 4 Department of Medicine, Joondalup Health Campus, Joondalup, WA 6027, Australia; 5 School of Surgery, University of Western Australia, Perth, WA 6009, Australia

**Keywords:** peripheral artery disease, drug-coated balloon, BIOtorcin, CRC-015, sirolimus, pharmacokinetics, superficial femoral artery, femoropopliteal disease, restenosis

## Abstract

**Background/Objectives:** Drug-coated balloons (DCBs) provide local antiproliferative therapy during endovascular revascularization without leaving a permanent vascular scaffold. Sirolimus is an attractive cytostatic antiproliferative agent, although its relatively low lipophilicity creates challenges for rapid arterial uptake and sustained tissue residence following balloon delivery. The BRight DCB is a novel peripheral DCB coated with BIOtorcin™ (CRC-015), a highly lipophilic synthetic derivative of sirolimus. The BRight PK study primarily evaluated the systemic pharmacokinetics of BIOtorcin™ and its metabolite sirolimus following treatment of femoropopliteal disease and secondarily assessed procedural performance, safety, patency, and functional outcomes over 12 months. **Methods:** BRight PK was a prospective, single-center, non-randomized pharmacokinetic study involving six adults with symptomatic peripheral artery disease (PAD; Rutherford–Becker category 2–4) caused by de novo superficial femoral and/or proximal popliteal artery disease. Following mandatory predilatation, lesions were treated with one or two BRight DCBs. Whole-blood BIOtorcin™ and sirolimus concentrations were measured before treatment and serially from 5 min to 24 h after removal of the final DCB. Non-compartmental pharmacokinetic analysis was performed. Angiography and duplex ultrasound were independently assessed by a core laboratory, and endpoint-related clinical events were independently adjudicated. **Results:** All six participants completed 12-month follow-up. Mean age was 70.8 ± 7.4 years, and four (66.7%) were male. Seven BRight DCBs treated six lesions. Mean core-laboratory lesion length was 140.85 ± 136.23 mm, and baseline diameter stenosis was 78.50 ± 23.39%. Mean BIOtorcin™ exposure was 3976.8 ± 1665.7 µg (0.051 ± 0.0188 mg/kg). BIOtorcin™ Cmax was 23.60 ± 9.88 ng/mL at 5 min, terminal half-life was 21.87 ± 10.17 h, AUC0–t was 28.30 ± 12.19 h·ng/mL, and AUC0–∞ was 47.47 ± 18.27 h·ng/mL. Sirolimus Cmax was 0.692 ± 0.462 ng/mL at 24 h, when concentrations remained on the ascending portion of the concentration–time curve, indicating that the 24 h sampling window captured only early systemic sirolimus exposure rather than a complete pharmacokinetic profile. Device success was 100% (7/7); core-laboratory technical and procedural success were each 66.7% (4/6). No deaths, major or minor amputations, clinically driven target lesion revascularizations (cd-TLR), or clinically driven target vessel revascularizations (cd-TVRs) occurred. Visit-based 12-month primary patency was 83.3% (5/6); the single binary restenosis was detected at day 391, resulting in Kaplan–Meier primary patency of 100% at calendar day 365. The mean overall Walking Impairment Questionnaire (WIQ) score increased from 21.87 ± 19.16 at baseline to 62.28 ± 38.78 at 12 months. **Conclusions:** BIOtorcin™ demonstrated rapid systemic appearance following BRight DCB treatment, followed by a progressive decline in circulating parent drug concentrations and low systemic exposure to its metabolite, sirolimus. These pharmacokinetic findings, together with successful device delivery and the absence of major safety concerns over 12 months, demonstrate procedural feasibility and support continued clinical evaluation of the BRight DCB. Given the pharmacokinetic study design and small sample size (*n* = 6), the 12-month clinical, functional, and patency findings are exploratory and hypothesis-generating and should not be interpreted as evidence of clinical efficacy.

## 1. Introduction

Peripheral artery disease (PAD) is a common manifestation of systemic atherosclerosis and affects more than 200 million people worldwide [1]. Lower-extremity PAD encompasses a clinical spectrum ranging from asymptomatic disease and intermittent claudication to ischemic rest pain and chronic limb-threatening ischemia. In addition to impairing walking capacity and quality of life, PAD is associated with increased risks of adverse limb events, cardiovascular morbidity, and mortality [1,2,3].

Endovascular revascularization has become an important component of contemporary treatment for symptomatic femoropopliteal disease. Percutaneous transluminal angioplasty can restore luminal diameter and blood flow without open surgical reconstruction; however, elastic recoil, vascular remodeling, and neointimal hyperplasia may result in restenosis following plain balloon angioplasty [4,5,6,7]. Bare-metal stents can improve acute luminal gain but introduce a permanent scaffold, with potential complications including stent fracture and in-stent restenosis [8].

DCBs were developed to combine mechanical angioplasty with local delivery of an antiproliferative agent while avoiding permanent implantation. Paclitaxel-coated balloons have demonstrated reductions in restenosis and repeat target lesion revascularization compared with uncoated balloon angioplasty across multiple randomized femoropopliteal trials [4,5,6,7,9,10,11,12]. Concerns raised in 2018 regarding a possible association between paclitaxel-coated peripheral devices and late mortality prompted extensive reassessment [13]. Subsequent randomized, patient-level, administrative, and real-world analyses did not reproduce this association [14,15,16,17,18,19], and the United States Food and Drug Administration subsequently concluded that the totality of available evidence did not support an excess mortality risk associated with paclitaxel-coated devices [20].

Interest has nevertheless continued in alternative antiproliferative agents and drug delivery technologies. Sirolimus is a potent inhibitor of vascular smooth muscle cell proliferation and migration. In contrast to paclitaxel, which exerts predominantly cytotoxic effects, sirolimus is principally cytostatic and has a broad therapeutic margin [21,22]. Sirolimus-based technologies have been extensively evaluated in coronary drug-eluting stents and increasingly in balloon-based applications [23,24,25,26,27]. However, conventional sirolimus has lower lipophilicity and less favorable tissue retention characteristics than paclitaxel, creating a challenge for balloon-based delivery in which drug transfer occurs during a relatively brief period of vessel wall contact [21,22].

The BRight DCB is an investigational peripheral DCB based on the Passeo-35 percutaneous transluminal angioplasty platform. The balloon is coated with BIOtorcin™ (CRC-015), a synthetic derivative of sirolimus reported to be approximately 1000-fold more lipophilic than sirolimus. BIOtorcin™ retains antiproliferative and antimigratory activity against vascular smooth muscle cells and was developed to improve rapid arterial uptake and tissue persistence after balloon delivery. The device carries approximately 1.8 µg/mm^2^ BIOtorcin™ over an underlayer of recombinant human serum albumin.

Preclinical pharmacokinetic studies provide biological support for this strategy. In a Good Laboratory Practice (GLP) porcine model, BIOtorcin™ remained measurable within femoral arterial tissue for up to 90 days after treatment with the BRight DCB. These observations demonstrate prolonged arterial tissue persistence in the experimental setting, although corresponding human arterial wall concentrations have not been directly measured.

The primary objective of the BRight PK study was therefore to characterize whole-blood concentrations and non-compartmental pharmacokinetic parameters of BIOtorcin™ and sirolimus following BRight DCB treatment of femoropopliteal atherosclerotic lesions. Secondary objectives were to characterize device and procedural performance and describe safety, primary patency, restenosis, revascularization, Rutherford category, resting ankle–brachial index (ABI), and patient-reported walking impairment over 12 months.

## 2. Materials and Methods

### 2.1. Study Design and Oversight

**Figure 1:**
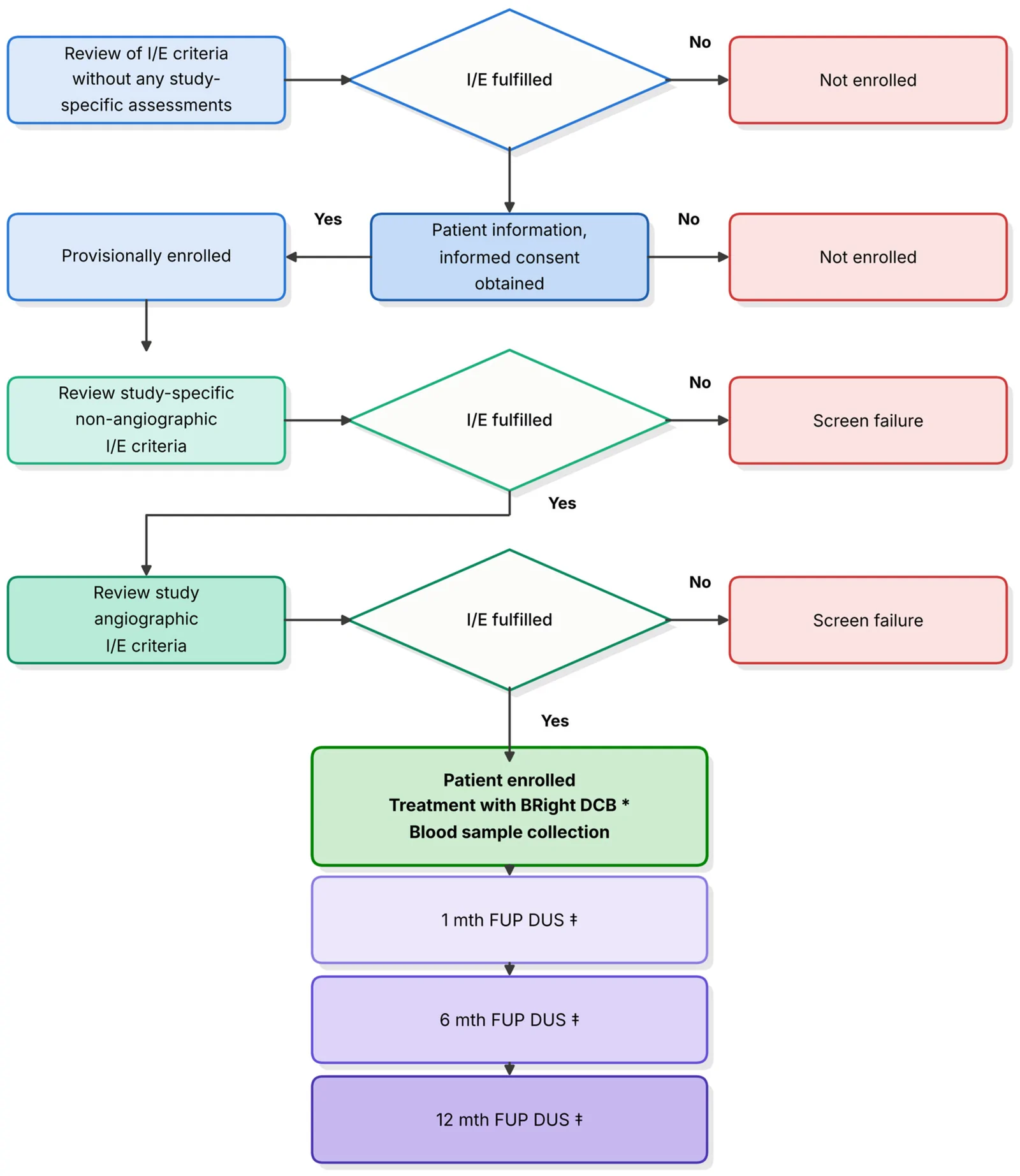
BRight PK study flow diagram. Sequential eligibility assessment, enrollment, BRight DCB treatment, pharmacokinetic blood sampling, and clinical/DUS follow-up through 12 months. *One or two BRight DCBs were permitted following mandatory predilatation. ‡Follow-up included clinical assessment and DUS. Abbreviations: DCB, drug-coated balloon; DUS, duplex ultrasound; FUP, follow-up; I/E, inclusion/exclusion; PK, pharmacokinetic.

BRight PK (ClinicalTrials.gov identifier NCT06065345) was a prospective, single-center, non-randomized pharmacokinetic clinical investigation conducted at Royal Perth Hospital, Perth, Western Australia. Six participants were enrolled between 30 April and 21 June 2024 and followed for 12 months. The final 12-month follow-up was completed on 22 July 2025. The study screening, enrolment, treatment, pharmacokinetic sampling, and follow-up pathway is summarized in Figure 1.

This study, entitled “BIOTRONIK—Pharmacokinetic Study of a Sirolimus Derivative-Coated Balloon (BRight DCB) in the Superficial Femoral and Proximal Popliteal Artery”, was approved under ethics reference RGS0000006363 on 16 August 2023. The investigation was conducted in accordance with the Declaration of Helsinki, ISO 14155 [28], International Council for Harmonisation Good Clinical Practice, and applicable regulatory requirements. Written informed consent was obtained before study-specific procedures.

An independent Clinical Events Committee adjudicated endpoint-related and potentially endpoint-related clinical events. Index procedure quantitative vascular angiography (QVA) and follow-up duplex ultrasound (DUS) examinations were independently analyzed by coreLab Black Forest GmbH (Bad Krozingen, Germany). Pharmacokinetic samples were analyzed by Frontage Laboratories, Inc. (Concord, OH, USA). Study monitoring incorporated 100% source data verification according to the monitoring plan.

### 2.2. Participants

Eligible participants were adults aged ≥18 years with lifestyle-limiting claudication or rest pain corresponding to Rutherford–Becker category 2, 3, or 4 and requiring treatment of a de novo lesion involving the superficial femoral artery (SFA) and/or proximal popliteal artery.

Principal angiographic inclusion criteria included a visually estimated reference vessel diameter of 5–6 mm, >50% target lesion stenosis, successful guidewire crossing, adequate inflow, patent P2 and P3 popliteal segments, and at least one patent infrapopliteal runoff vessel in continuity with the native femoropopliteal artery. Successful predilatation was required before study device treatment.

Major exclusions included previous endovascular treatment of the target lesion, in-stent restenosis, acute or subacute target vessel thrombus, aneurysmal target vessel, chronic renal insufficiency with estimated glomerular filtration rate <30 mL/min, renal replacement therapy, inability to walk without assistance, immunosuppressive therapy, and requirement for adjunctive atherectomy, laser, cryoplasty, scoring/cutting balloon, or brachytherapy.

### 2.3. Investigational Device

The investigational device is the BRight DCB (Passeo-35 coated with BIOtorcin™) and was available in four sizes: 5 × 80 mm, 5 × 120 mm, 6 × 80 mm, and 6 × 120 mm. The BRight DCB is intended for dilatation of de novo lesions in native superficial femoral or popliteal arteries, with simultaneous release of BIOtorcin™ to the vessel wall as a secondary action intended to reduce restenosis of the treated vessel segment. The BRight DCB is currently an investigational device.

**Figure 2:**
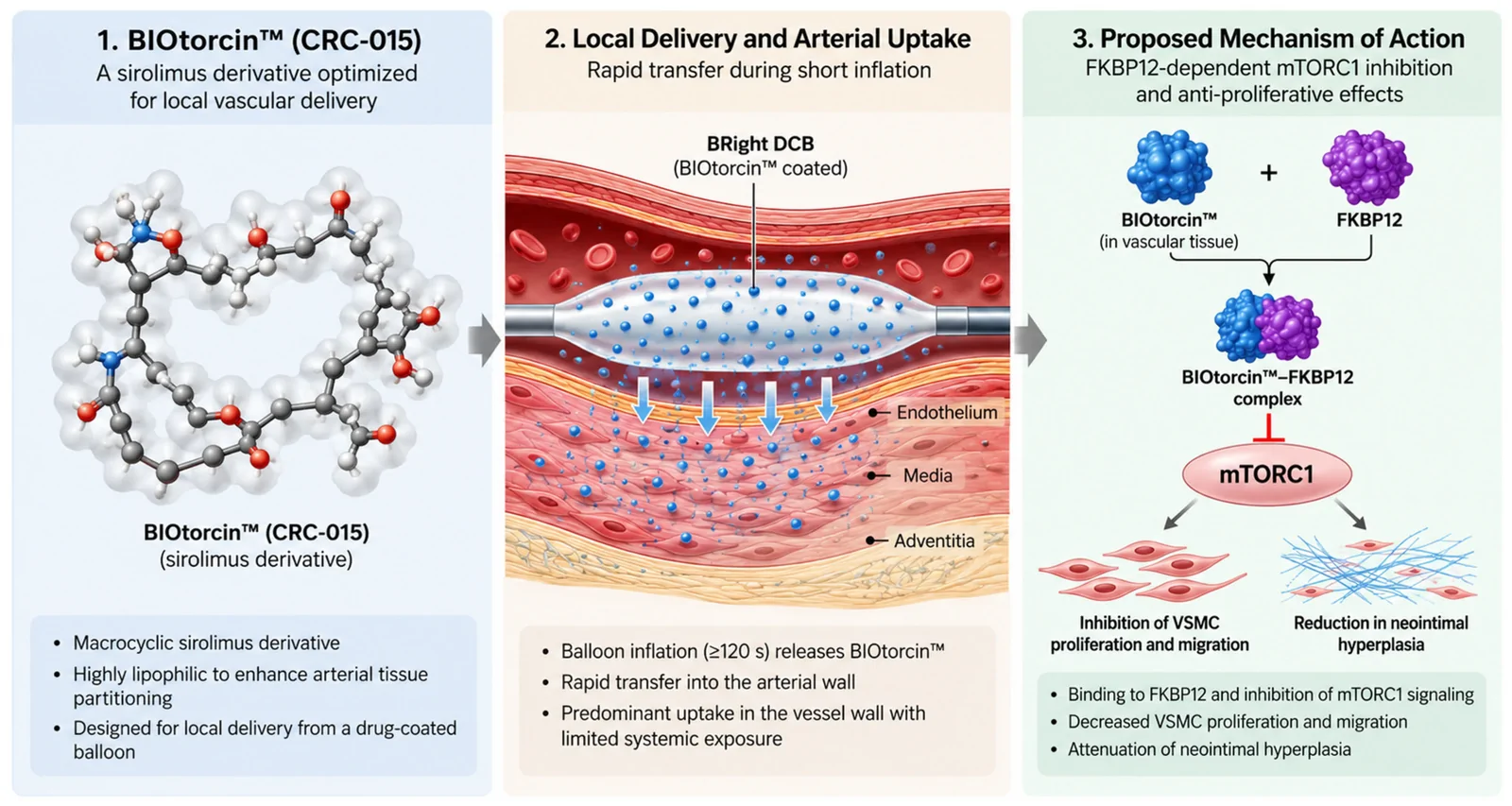
BIOtorcin™ (CRC-015): local arterial delivery and proposed mechanism of action. BIOtorcin™ is a highly lipophilic sirolimus derivative delivered locally from the BRight drug-coated balloon during balloon inflation. Following arterial uptake, its proposed pharmacological action involves FKBP12-dependent inhibition of mTORC1 signaling, with consequent suppression of vascular smooth muscle cell proliferation and migration and attenuation of neointimal hyperplasia. The pathway shown is schematic and represents the proposed mechanism of action. Abbreviations: DCB, drug-coated balloon; FKBP12, FK506-binding protein 12; mTORC1, mechanistic target of rapamycin complex 1; VSMC, vascular smooth muscle cell.

The BIOtorcin™-coated PTA balloon catheter is manufactured by applying an underlayer of recombinant human serum albumin (HSA) to an uncoated Passeo-35 balloon catheter and then applying the BIOtorcin™ drug coating. The principal components are the Passeo-35 balloon catheter (non-hydrophobic coating version; BIOTRONIK), 5% Recombumin^®^ Prime cGMP HSA as the underlayer, and BIOtorcin™ (CRC-015), a derivative of rapamycin/sirolimus, as the active drug substance. The local delivery of BIOtorcin™ from the BRight DCB and its proposed sirolimus-related mechanism of action are illustrated schematically in Figure 2.

The Passeo-35 platform has two radiopaque markers at the balloon ends for fluoroscopic visualization and positioning and two proximal Luer ports for balloon inflation/deflation and guidewire/lumen access/flushing. The catheter has a usable length of 130 cm and is compatible with 0.035-inch guidewires. For BRight PK, only the BIOTRONIK AG (Bülach, Switzerland) 6F Fortress^®^ introducer sheath was permitted.

The BIOtorcin™ drug substance layer consists of BIOtorcin™ at 100 wt%, with a nominal drug load of approximately 1.8 µg/mm^2^ of the outer balloon surface area, including the cylindrical section and half of both cones. The recombinant HSA underlayer is applied beneath the drug layer and is not part of the drug substance layer. BIOtorcin™ is a potent lipophilic derivative of sirolimus and is reported to be approximately 1000-fold more lipophilic than sirolimus. It is designed to inhibit proliferation and migration of vascular smooth muscle cells and thereby inhibit restenosis. The recombinant HSA underlayer is produced under cGMP and, according to the clinical investigation documentation, is expressed by fermentation of Saccharomyces cerevisiae without animal- or human-derived source materials. The study device was stored in a dry location protected from sunlight at 15–25 °C; short-term excursions between 10 °C and 40 °C were permitted.

### 2.4. Index Procedure

Femoral access was mandatory. The target lesion was crossed with a 0.035-inch guidewire. Predilatation with an uncoated PTA balloon was required. Successful predilatation was defined by the protocol as residual stenosis ≤30% without flow-limiting dissection.

The BRight DCB diameter was selected to correspond to the distal reference vessel diameter. The balloon was intended to cover the entire treated lesion with approximately 10 mm margins proximally and distally. A maximum of two BRight DCBs was permitted. When two devices were required, an overlap of at least 10 mm was recommended. Each DCB was gradually inflated for a minimum of 120 s.

Post-dilatation with an uncoated balloon was permitted at investigator discretion. Bailout nitinol stenting was permitted for residual stenosis >30% or major flow-limiting dissection. All participants received standard post-procedural dual antiplatelet therapy, consisting of aspirin indefinitely and clopidogrel for approximately 1–3 months, per institutional practice.

### 2.5. Pharmacokinetic Sampling and Bioanalysis

Approximately 5 mL of venous whole blood was collected before balloon treatment and at approximately 5, 15, and 30 min and 1, 2, 3, and 8 h after removal of the final BRight DCB. A 24 h sample was collected when feasible; this sampling window was the protocol-specified limit for this first-in-human feasibility pharmacokinetic study and was considered sufficient to characterize the primary objective of early systemic BIOtorcin™ exposure, although it was not designed to capture a complete terminal elimination profile for sirolimus (Section 4.8). Samples were collected into K3-EDTA tubes and processed, preserved, frozen, and transported according to the study bioanalytical protocol. Whole-blood concentrations of BIOtorcin™ and sirolimus were measured using validated liquid chromatography–tandem mass spectrometry methods.

### 2.6. Pharmacokinetic Endpoints

Non-compartmental pharmacokinetic analysis was performed using Phoenix WinNonlin version 8.3.5.340. Prespecified parameters included maximum observed concentration (Cmax), time to maximum observed concentration (Tmax), area under the concentration–time curve from zero to the last measurable concentration (AUC_0_–t), area under the concentration–time curve extrapolated to infinity (AUC_0_–∞), apparent terminal elimination rate constant (λz), terminal elimination half-life (t½), apparent total clearance, and apparent terminal volume of distribution when estimable.

AUC_0_–t was calculated using linear trapezoidal summation. AUC_0_–∞ was calculated as AUC_0_–t + Ct/λz, where Ct was the final measurable concentration. Terminal half-life was calculated as ln(2)/λz. Cmax and Tmax were determined directly from the observed concentration–time data without interpolation.

### 2.7. Clinical Endpoints

Device success was defined as successful delivery, inflation/deflation, and retrieval of the intact study device. Acute technical success required successful vascular access and completion of the endovascular procedure, immediate final residual diameter stenosis ≤30% by independent core-laboratory QVA, and absence of bailout stenting. Acute procedural success required technical success without death, major target limb amputation, target lesion thrombosis, or cd-TLR within 72 h.

Major adverse events (MAE) comprised device- or procedure-related death within 30 days, major target limb amputation, or cd-TLR. Binary restenosis was defined as DUS peak systolic velocity ratio (PSVR) >2.5 or angiographic stenosis >50% based on QVA. Primary patency was defined as DUS PSVR ≤2.5 or angiographic stenosis ≤50% together with absence of cd-TLR. Rutherford category, resting target limb ABI, and WIQ were assessed longitudinally. Standardized cardiovascular endpoint definitions and PACSS assessment were used where applicable [29,30].

### 2.8. Statistical Analysis

The primary statistical objective was descriptive characterization of BIOtorcin™ and sirolimus pharmacokinetics. No formal hypothesis was tested. The sample size was feasibility-based and was considered adequate for the primary pharmacokinetic objective.

The pharmacokinetic analysis set comprised participants in whom an investigational BRight DCB entered the introducer sheath following successful predilatation, who had at least one blood sample before DCB deployment and one after removal of the final DCB, and who had an evaluable concentration–time profile. The full analysis set included all enrolled participants in whom an investigational DCB entered the introducer sheath following successful predilatation. Safety analyses were based on the full analysis set.

For quantitative variables, mean, SD, median, minimum, maximum, and 95% CI for the mean were calculated where specified in the statistical analysis plan. Categorical variables were summarized as absolute and relative frequencies, with exact 95% CIs for proportions when relevant. Pharmacokinetic parameters were summarized descriptively. No missing PK values were imputed. An isolated non-critical missing sample did not exclude a participant when the remaining concentration–time profile remained evaluable.

Clinical secondary time-to-event endpoints—including MAE, mortality, major and minor amputation, cd-TLR, cd-TVR, binary restenosis, and primary patency—were evaluated using Kaplan–Meier methods. Because of the deliberately small sample size and exploratory nature of secondary clinical endpoints, no formal repeated-measures hypothesis testing, subgroup analyses, multiplicity correction, or multivariable modeling was performed. Clinical and functional findings were interpreted descriptively.

## 3. Results

### 3.1. Study Enrollment, Analysis Population, and Follow-Up

Six participants with symptomatic femoropopliteal PAD were enrolled at Royal Perth Hospital between 30 April and 21 June 2024. All six underwent treatment with at least one BRight DCB and were included in the full analysis and pharmacokinetic analysis sets. All participants completed the scheduled 1-, 6-, and 12-month follow-up visits, corresponding to 100% follow-up completeness. There were no deaths, withdrawals, losses to follow-up, or other early terminations (Figure 1).

Pharmacokinetic sampling was highly complete. Whole-blood samples were obtained from all six participants before treatment and at 5 min, 15 min, 30 min, 1 h, 2 h, and 24 h after removal of the final BRight DCB. Four participants had an available 3 h sample and five had an available 8 h sample. Missing pharmacokinetic observations were not imputed, and the isolated missing samples did not preclude pharmacokinetic characterization.

### 3.2. Participant Characteristics

Mean age was 70.8 ± 7.39 years (range, 61–83 years), and four participants (66.7%) were male. Mean height was 172.83 ± 7.83 cm, mean body weight was 78.67 ± 19.64 kg, and mean body mass index was 26.13 ± 5.10 kg/m^2^.

Hypertension was present in four participants (66.7%), diabetes mellitus in three (50.0%), hyperlipidemia in three (50.0%), and coronary artery disease in three (50.0%). One participant (16.7%) had cerebrovascular disease and one (16.7%) had renal disease without dialysis. Three participants (50.0%) had a recorded history of peripheral arterial occlusive disease, and two (33.3%) had undergone previous peripheral vascular intervention and/or surgery. All six had a smoking history; four (66.7%) were current smokers and two (33.3%) were former smokers. At baseline, one participant was Rutherford category 2, three were category 3, and two were category 4 (Table 1).

### 3.3. Baseline Laboratory Characteristics

Mean estimated glomerular filtration rate was 78.3 ± 17.96 mL/min (range, 46–90 mL/min). One participant had an eGFR outside the local laboratory reference range, judged not clinically significant. Mean platelet count was 241.3 ± 44.73 × 10^9^/L and mean white blood cell count was 7.703 ± 1.569 × 10^9^/L. No participant had an abnormal platelet or white blood cell count according to the study laboratory reference ranges.

### 3.4. Target Lesion Characteristics

Six target lesions were treated. All involved the SFA, and based on independent core-laboratory assessment, two extended into the popliteal artery. Four lesions (66.7%) were stenotic and two (33.3%) were occluded. Investigator-assessed TASC classification was type A in two lesions, type B in three, and type C in one. No target lesion thrombus was identified.

Mean investigator-reported lesion length was 72.7 ± 38.09 mm, whereas independent core-laboratory mean lesion length was 140.85 ± 136.23 mm (range, 14.43–378.73 mm); the core-laboratory measurement was used as the principal lesion length result. Mean core-laboratory reference vessel diameter was 5.527 ± 0.409 mm and baseline diameter stenosis was 78.50 ± 23.39%. Among the two occlusions, mean occlusion length was 62.15 ± 30.35 mm. Calcific burden was substantial: one lesion was PACSS grade 0, one grade 1, two grade 3, and two grade 4; thus, four of six lesions (66.7%) were PACSS grade 3–4 (Table 2).

### 3.5. Procedural Characteristics and Quantitative Angiography

Predilatation was performed in all six target lesions. Seven BRight DCBs were subsequently deployed; five participants received one DCB and one received two. Five of seven study balloons were 120 mm long and two were 80 mm; five were 6 mm and two were 5 mm in diameter. Mean maximum balloon pressure was 8.3 ± 1.80 atm and mean DCB inflation duration was 207.4 ± 37.41 s (range, 180–272 s). Two lesions underwent post-dilatation. No bailout stent was implanted.

Core-laboratory QVA showed an increase in mean minimum lumen diameter from 1.248 ± 1.368 mm before treatment to 3.645 ± 0.487 mm after predilatation and 3.963 ± 0.594 mm after completion of treatment. Mean residual stenosis was 34.08 ± 6.96% after predilatation and 28.49 ± 7.64% after completion of treatment. Investigators reported only grade A or B dissections, whereas the core laboratory classified four of six lesions as grade D after completion of treatment; one lesion had no dissection and one was not assessable. Despite these findings, no bailout stenting was required.

Device success was achieved for all seven BRight DCBs (100%). Core-laboratory-defined acute technical and acute procedural success were each achieved in four of six cases (66.7%). No index limb embolic event occurred (Table 2).

### 3.6. BIOtorcin™ Exposure

Mean total BIOtorcin™ dose delivered per participant was 3976.8 ± 1665.7 µg (range, 2761–7205 µg), corresponding to a body weight-normalized mean dose of 0.051 ± 0.0188 mg/kg (range, 0.037–0.088 mg/kg). Five participants received a single DCB and one participant received two 120 mm balloons, resulting in the highest exposure of 7205 µg (0.088 mg/kg). Participant-level analyses showed a low proportion of drug remaining on the balloon after deployment, consistent with substantial release from the device. This balloon-residual assessment reflects drug remaining on the device after withdrawal and does not separately quantify any drug loss that may occur during introduction through the sheath or transit to the target lesion; such transit loss was not specifically measured in this study (Section 4.8). Detailed participant-level exposure is provided in Supplementary Table S1.

### 3.7. Primary Endpoint: BIOtorcin™ Pharmacokinetics

BIOtorcin™ appeared rapidly in the systemic circulation. The maximum observed whole-blood concentration occurred at the first post-treatment sampling point, 5 min after removal of the final DCB, in all six participants. Mean BIOtorcin™ Cmax was 23.60 ± 9.88 ng/mL (range, 10.50–36.50 ng/mL), with a uniform Tmax of 0.08 h. The principal BIOtorcin™ pharmacokinetic parameters are summarized in Table 3 and Supplementary Figure S1.

Following this early peak, systemic BIOtorcin™ concentrations decreased progressively and were approximately 0.60 ± 0.23 ng/mL at 24 h. Mean AUC_0_–t was 28.30 ± 12.19 h·ng/mL and mean AUC_0_–∞ was 47.47 ± 18.27 h·ng/mL. Mean apparent terminal elimination rate constant was 0.0383 ± 0.0172 h^−1^, corresponding to a mean terminal half-life of 21.87 ± 10.17 h (range, 12.23–36.43 h) (Supplementary Figure S1; Table 3).

### 3.8. Sirolimus Pharmacokinetics

**Figure 3:**
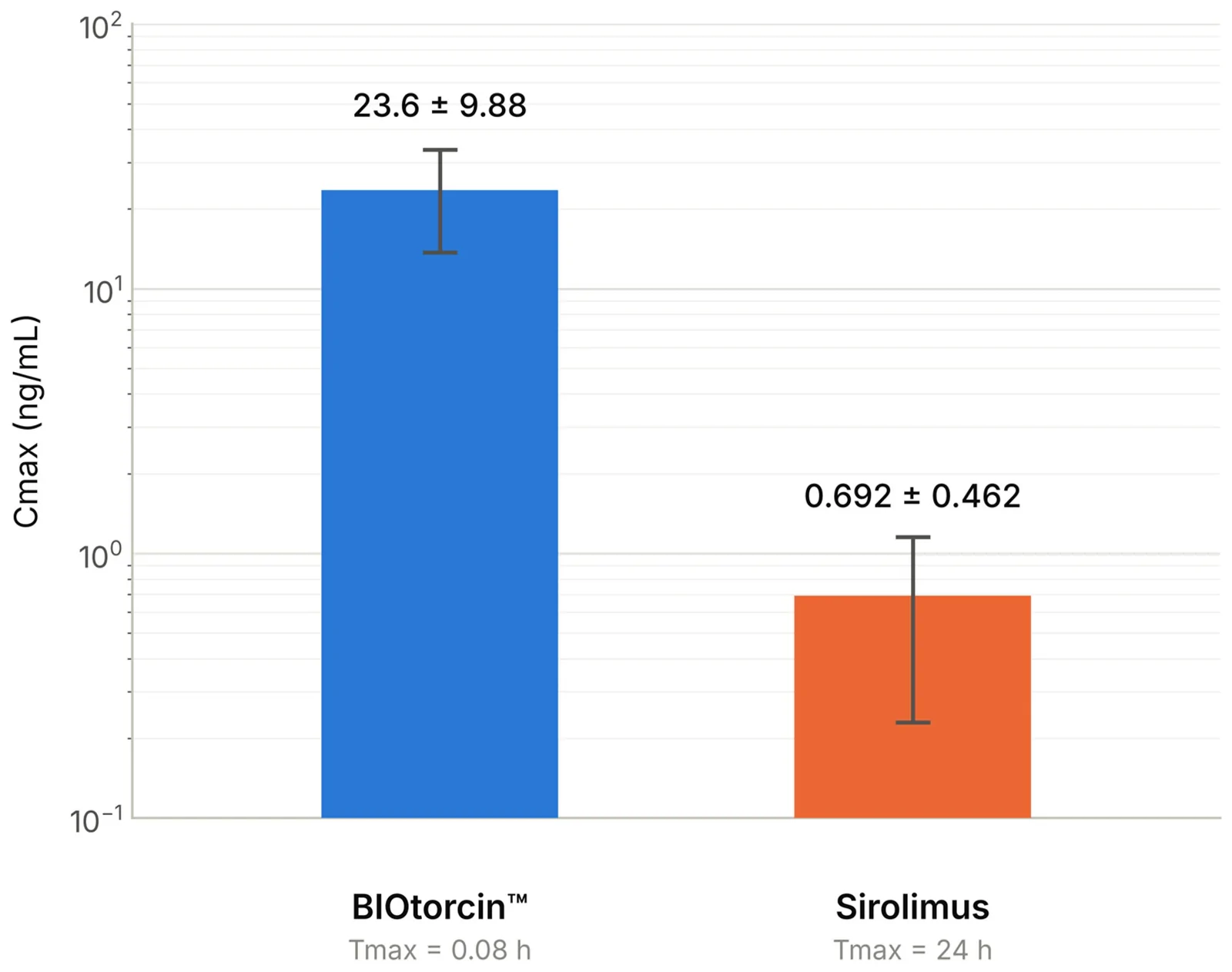
Peak systemic exposure to BIOtorcin™ and sirolimus. Maximum observed whole-blood concentrations (Cmax; mean ± SD) following BRight DCB treatment (*n* = 6), displayed on a logarithmic scale. BIOtorcin™ peaked at 5 min, whereas sirolimus concentrations remained increasing at the final 24 h sampling point. Abbreviations: Cmax, maximum observed concentration; SD, standard deviation.

The systemic concentration–time profile of sirolimus differed from that of BIOtorcin™. Sirolimus concentrations began increasing approximately 30 min after removal of the final DCB and continued to rise through the final 24 h sampling point. Mean sirolimus Cmax was 0.692 ± 0.462 ng/mL (range, 0.25–1.57 ng/mL), with Tmax of 24 h in all participants. Mean AUC_0_–t was 9.307 ± 6.026 h·ng/mL. Comparative peak systemic exposure to BIOtorcin™ and sirolimus is shown in Figure 3, with pharmacokinetic parameters summarized in Table 3.

Because sirolimus concentrations remained on the ascending portion of the concentration–time curve at 24 h, a terminal elimination phase could not be adequately characterized; consequently, AUC_0_–∞, λz, and t½ were not estimated for sirolimus (Figure 3; Table 3).

### 3.9. Periprocedural Performance

Device success was 7/7 (100%). Acute technical success was achieved in four of six lesions (66.7%), and acute procedural success was achieved in four of six participants (66.7%). The two cases that did not satisfy the technical success definition reflected the stringent protocol requirement for final residual stenosis ≤30% by independent QVA rather than failure of device delivery or retrieval. No bailout stent was implanted and no angiographically significant index limb embolic event occurred.

### 3.10. Major Adverse Events, Survival, Limb Outcomes, and Revascularization

Freedom from the prespecified MAE composite was 100% over 12 months. No participant died during the study. There were no major or minor index limb amputations. No participant underwent cd-TLR or cd-TVR, resulting in 100% freedom from both revascularization endpoints over 12 months. All potentially endpoint-related clinical events were subject to independent Clinical Events Committee adjudication (Table 4).

### 3.11. Binary Restenosis and Primary Patency

No binary restenosis was identified at 6 months. One participant demonstrated binary restenosis on independent core-laboratory duplex assessment during the protocol-defined 12-month follow-up window, yielding a visit-based binary restenosis rate of 16.7% (1/6). Primary patency was present in all six lesions at 6 months and in five of six at the 12-month visit.

The single loss of patency was documented 391 days after the index procedure, beyond calendar day 365 but within the protocol-permitted 12-month visit window. Consequently, Kaplan–Meier primary patency at exactly day 365 was 100%, whereas observed visit-based 12-month primary patency was 83.3% (5/6). No cd-TLR occurred in association with the duplex-defined restenosis (Table 4).

### 3.12. Rutherford Clinical Category

At baseline, one participant was Rutherford category 2, three were category 3, and two were category 4. At 1 month, four participants were category 0, one category 1, and one category 3. At 6 months, three were category 0, one category 1, and two category 2. At 12 months, three were category 0, two category 2, and one category 4. Five of six participants had a lower Rutherford category at 12 months than at baseline.

Mean change in Rutherford category from baseline was −2.50 ± 0.837 at 1 month, −2.33 ± 1.211 at 6 months, and −1.83 ± 1.941 at 12 months. Detailed distributions are provided in Supplementary Table S2.

### 3.13. Ankle–Brachial Index

Mean resting target limb ABI was 0.723 ± 0.1418 at baseline (95% CI, 0.5745–0.8721), 0.880 ± 0.1349 at 1 month (95% CI, 0.7384–1.0216), 0.734 ± 0.0999 at 6 months (*n* = 5; 95% CI, 0.6100–0.8580), and 0.752 ± 0.2047 at 12 months (95% CI, 0.5369–0.9655). The paired mean change from baseline was +0.16 ± 0.135 at 1 month, +0.01 ± 0.116 at 6 months, and +0.03 ± 0.194 at 12 months. At baseline, three participants had moderate-range ABI, two mild-range ABI, and one normal ABI; at 1 month, three had mild-range and three normal ABI; at 6 months, one had moderate-range and four mild-range ABI; and at 12 months, two participants were in each of the moderate, mild, and normal categories. The largest mean hemodynamic improvement was observed at 1 month and was not sustained to the same magnitude at later follow-up (Table 5; Supplementary Table S3).

### 3.14. Walking Impairment Questionnaire

**Figure 4:**
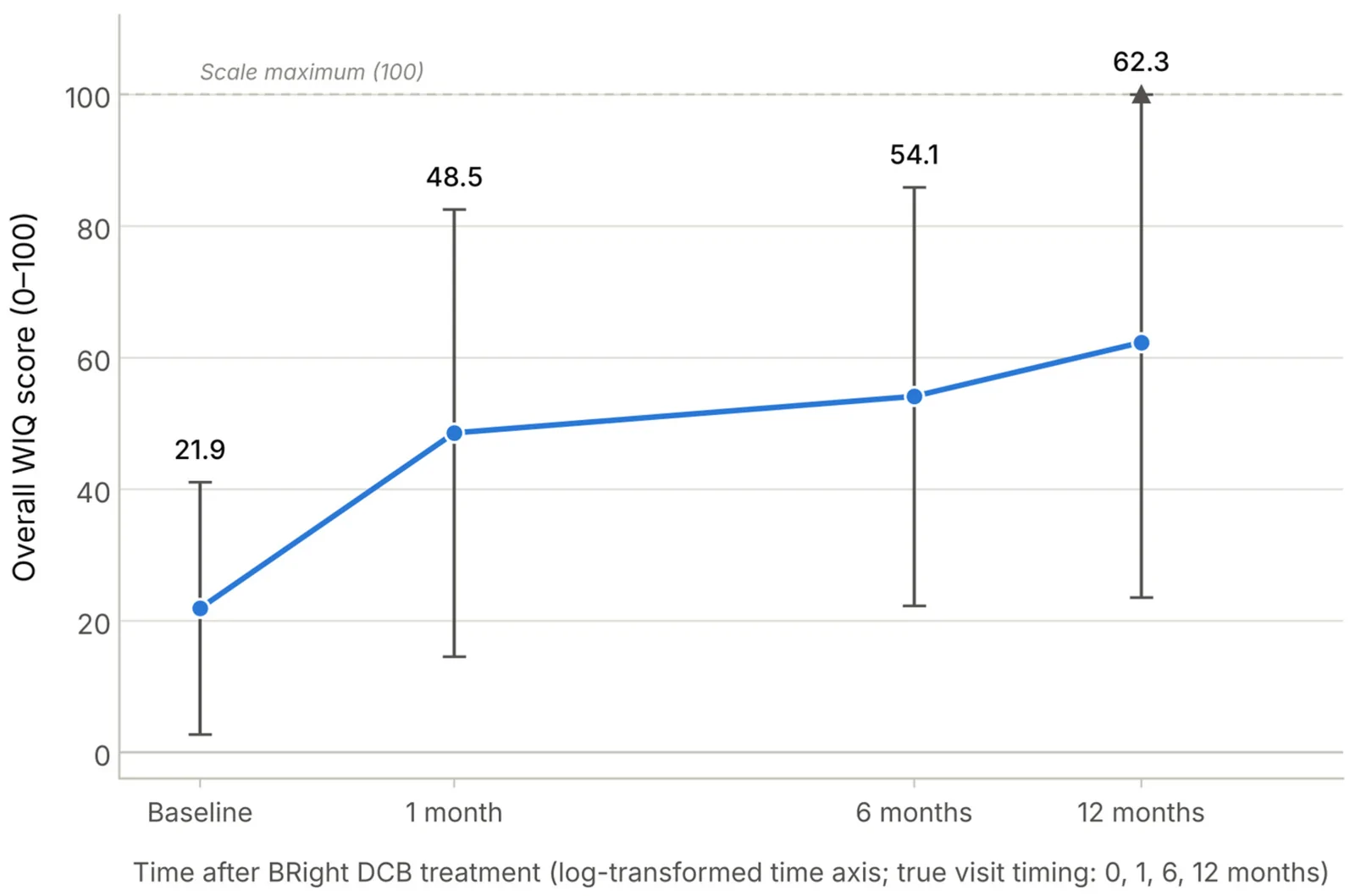
Walking Impairment Questionnaire scores through 12 months. Mean ± SD overall WIQ scores are shown at baseline and at 1, 6, and 12 months after BRight DCB treatment, plotted against a log-transformed time axis to preserve readability given the uneven visit spacing (tick positions reflect true visit timing). Because the WIQ is bounded 0–100, the upper error bar at 12 months is truncated at the scale maximum (uncensored mean + SD = 101.1). Higher scores indicate better self-reported walking function. Results are descriptive given the small sample size (*n* = 6). Abbreviations: SD, standard deviation; WIQ, Walking Impairment Questionnaire.

Patient-reported walking function improved numerically following treatment. Mean overall WIQ score increased from 21.87 ± 19.16 at baseline to 48.53 ± 33.96 at 1 month, 54.08 ± 31.81 at 6 months, and 62.28 ± 38.78 at 12 months. Longitudinal WIQ results are shown in Figure 4 and Table 5.

Improvements were observed across all WIQ domains. At 12 months, mean pain score was 62.50 ± 41.08 versus 33.33 ± 25.82 at baseline; walking distance score was 71.50 ± 44.68 versus 42.83 ± 29.63; walking speed score was 55.62 ± 38.56 versus 17.93 ± 18.60; and stair-climbing score was 59.72 ± 40.02 versus 4.86 ± 10.01. Mean paired change in overall WIQ was +26.66 ± 26.41 at 1 month, +32.21 ± 37.63 at 6 months, and +40.40 ± 40.41 at 12 months (Figure 4; Table 5; Supplementary Table S4).

### 3.15. Adverse Events and Device Safety

No device deficiencies were reported. A total of 13 adverse events occurred among the six participants during 12-month follow-up. Four serious adverse events occurred in two participants. Two serious events were gastrointestinal disorders and two were categorized as other events, including bone fractures. There were no serious adverse device effects, unanticipated serious adverse device effects, or anticipated serious adverse device effects. No pattern of serious toxicity attributable to BIOtorcin™ or sirolimus was identified. A detailed adverse event summary is provided in Supplementary Table S5.

## 4. Discussion

### 4.1. Principal Findings

The BRight PK study provides the first dedicated clinical characterization of systemic BIOtorcin™ exposure following treatment of femoropopliteal atherosclerotic lesions with the BRight DCB. BIOtorcin™ reached its maximum observed whole-blood concentration rapidly after treatment, at 5 min following removal of the final DCB, and subsequently declined, whereas its metabolite sirolimus appeared later and remained on an ascending concentration–time trajectory at the final 24 h sampling point. These pharmacokinetic observations were accompanied by 100% device success and by the absence of death, major or minor amputation, clinically driven target lesion revascularization, clinically driven target vessel revascularization, or major adverse events over 12 months.

### 4.2. Pharmacological Rationale for BIOtorcin™

Sirolimus is a potent inhibitor of vascular smooth muscle cell proliferation and migration, biological processes that contribute importantly to neointimal hyperplasia following arterial injury [21,22]. Its predominantly cytostatic mechanism distinguishes sirolimus from paclitaxel and has driven extensive development of limus-based coronary drug-eluting technologies [21,31,32,33,34,35].

The application of conventional sirolimus to balloon-based delivery is more challenging because rapid transfer must occur during a brief period of arterial wall contact. The relatively limited lipophilicity of conventional sirolimus compared with paclitaxel can reduce immediate tissue partitioning and persistence [21,22]. This has stimulated the development of specialized sirolimus-coated balloon technologies using nanoparticles, phospholipid carriers, micro-reservoirs, and sustained-release platforms [23,24,25,26,27].

BIOtorcin™ represents a different pharmacological approach. Rather than relying exclusively on a delivery carrier to modify tissue kinetics, BIOtorcin™ is itself a synthetic, highly lipophilic derivative of sirolimus. The rationale is to retain the antiproliferative and antimigratory properties associated with sirolimus while enhancing rapid vessel wall uptake and tissue persistence.

### 4.3. BIOtorcin™ Pharmacokinetics and Preclinical Tissue Persistence

The principal pharmacokinetic finding was the rapid appearance and subsequent decline of BIOtorcin™ in systemic circulation. Mean Cmax was 23.60 ± 9.88 ng/mL and occurred at the first post-treatment sampling point, 5 min after removal of the final DCB. Mean AUC_0_–t was 28.30 ± 12.19 h·ng/mL, AUC_0_–∞ was 47.47 ± 18.27 h·ng/mL, and terminal half-life was 21.87 ± 10.17 h. This concentration–time profile is consistent with rapid release during balloon treatment followed by progressive systemic clearance.

**Figure 5:**
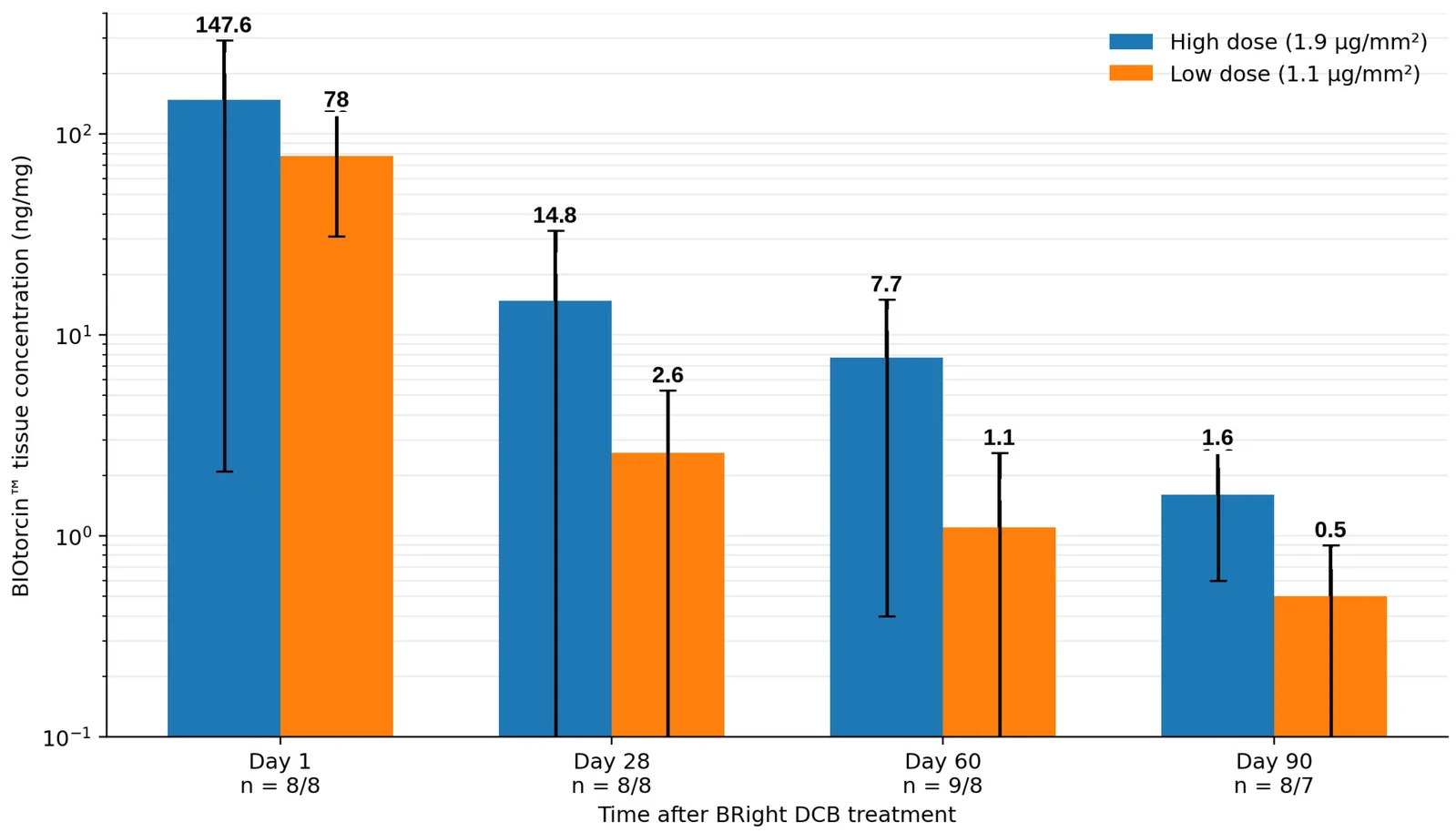
Preclinical BIOtorcin™ retention in porcine femoral arteries. Mean ± SD arterial tissue concentrations are shown after BRight DCB treatment with high-dose (1.9 µg/mm^2^) and low-dose (1.1 µg/mm^2^) BIOtorcin™ formulations. BIOtorcin™ remained measurable through 90 days in both groups. Data are from a preclinical GLP porcine study and do not represent human BRight PK tissue measurements. Abbreviations: DCB, drug-coated balloon; GLP, Good Laboratory Practice; SD, standard deviation.

Preclinical GLP studies demonstrated that BIOtorcin™ remained measurable within porcine femoral arterial tissue through 90 days after BRight DCB treatment. With the 1.9 µg/mm^2^ formulation, mean tissue concentrations were 147.6 ng/mg at day 1, 14.8 ng/mg at day 28, 7.7 ng/mg at day 60, and 1.6 ng/mg at day 90. Corresponding concentrations after the 1.1 µg/mm^2^ formulation were 78.0, 2.6, 1.1, and 0.5 ng/mg (Figure 5).

These preclinical data provide mechanistic support for prolonged arterial tissue persistence. However, human arterial tissue concentrations were not measured in BRight PK. The human clinical study therefore demonstrates systemic pharmacokinetics, whereas the porcine study provides preclinical tissue pharmacokinetics. The two datasets are complementary but should not be conflated. Rapid systemic appearance and subsequent decline of BIOtorcin™ in whole blood cannot, by themselves, demonstrate efficient arterial uptake or prolonged tissue retention in humans; a comparable systemic profile could in principle arise even from a drug taken up less efficiently by the vessel wall but cleared more slowly from the circulation. Accordingly, the BRight PK systemic pharmacokinetic findings should be interpreted as being consistent with, rather than as direct evidence for, efficient local drug delivery, and statements linking them to local pharmacological efficiency should be appropriately qualified. Confirmation of the proposed pharmacological advantage of BIOtorcin™—specifically, efficient arterial uptake and prolonged human tissue persistence—will ultimately require direct human arterial tissue sampling or other validated approaches capable of demonstrating local drug persistence, rather than inference from systemic concentrations alone.

### 4.4. Sirolimus Formation and Systemic Exposure

The systemic concentration–time profile of sirolimus was clearly different from that of BIOtorcin™. Sirolimus concentrations began increasing later and continued to rise over 24 h. Mean Cmax was 0.692 ± 0.462 ng/mL and the highest individual observed concentration was 1.57 ng/mL. Because concentrations remained on the ascending portion of the curve at 24 h, terminal sirolimus pharmacokinetic parameters could not be reliably estimated.

For clinical context, the observed concentrations were substantially below concentrations encountered during systemic sirolimus treatment. The therapeutic drug-monitoring literature demonstrates substantially higher trough concentration ranges during systemic sirolimus therapy [36,37,38]. Nevertheless, comparison with chronic systemic therapy should be made cautiously because the route of administration, treatment duration, indication, and pharmacokinetic exposure are fundamentally different. The low observed systemic concentrations therefore provide reassuring pharmacological context but should not be interpreted as defining a formal safety threshold for local DCB administration.

### 4.5. Peripheral Sirolimus-Coated Balloon Development

Clinical interest in limus-based DCBs for PAD has increased substantially. The XTOSI pilot study evaluated a sirolimus-coated balloon in femoropopliteal and below-the-knee arterial disease and reported encouraging clinical outcomes over 12 months [23]. The first-in-human SELUTION sustained limus release study similarly demonstrated the feasibility of limus-based DCB treatment in femoropopliteal lesions [24]. More recently, the SELUTION SFA Japan trial reported favorable outcomes following treatment of femoropopliteal lesions with a sirolimus-coated balloon [25]. Contemporary systematic reviews and observational studies continue to support investigation of limus-coated technologies across different peripheral arterial territories [26,27,39].

Direct numerical comparison between BRight PK and these studies is inappropriate. BRight PK included only six participants and was specifically designed to characterize pharmacokinetics rather than establish comparative clinical efficacy. Nevertheless, these studies provide important context for the broader development of limus-based balloon therapy.

### 4.6. Procedural Performance

The lesions treated in BRight PK were frequently complex. Mean core-laboratory lesion length was 140.85 mm, two lesions were occlusions, and four of six lesions were classified as PACSS grade 3 or 4. Peripheral arterial calcification is known to increase procedural complexity and may adversely affect luminal expansion, drug transfer, and subsequent patency [30].

Despite these characteristics, all seven BRight DCBs were successfully delivered, inflated, deflated, and retrieved. No bailout stenting was required and no index limb embolic event occurred. Core-laboratory-defined technical success and procedural success were both 66.7%. These results principally reflected the protocol requirement for final residual stenosis ≤30%, rather than device malfunction or inability to complete treatment.

Independent core-laboratory review identified grade D dissections in four lesions, whereas on-site investigators reported only grade A or B dissections in the same cases. Although striking, this magnitude of discrepancy is consistent with previously described differences between investigator and core-laboratory dissection grading in peripheral and coronary balloon angioplasty, which typically reflect differences in viewing conditions, magnification, use of multiple angiographic projections, and the systematic, blinded application of a standardized classification system by a dedicated core laboratory, as opposed to a single intraprocedural visual assessment by the treating investigator. Angiographic dissection classification has traditionally been used to characterize vessel wall injury after balloon angioplasty [40].

Critically, none of the grade D dissections triggered bailout stenting. Under the study protocol, bailout nitinol stenting was mandated for major flow-limiting dissection or residual stenosis >30% (Section 2.4); because none of these lesions met the bailout threshold, the treating investigators judged the dissections to be non-flow-limiting on the basis of preserved antegrade angiographic flow and acceptable residual stenosis at the end of the procedure, rather than by a separate, prespecified hemodynamic or pressure-gradient assessment. This real-time, qualitative judgment is itself a limitation, and the marked discrepancy with the independent core-laboratory grade D classification raises the possibility that the true severity of vessel wall injury was underappreciated at the time of treatment. Whether these dissections influenced subsequent patency cannot be determined with confidence in six participants; the single binary restenosis in this cohort occurred at day 391, well outside the acute post-procedural period in which dissection-related flow compromise would most likely manifest, which argues against a direct causal relationship, but the sample size precludes a definitive conclusion. Closer surveillance or more systematic hemodynamic assessment of high-grade dissections, even when angiographically non-flow-limiting, may be warranted in future, larger BRight DCB studies.

The substantial discrepancy between investigator-reported mean lesion length (72.7 ± 38.09 mm) and independent core-laboratory measurement (140.85 ± 136.23 mm) likely reflects similar methodological differences: investigator estimates were made visually during the procedure without calibrated quantitative vascular angiography, whereas the core laboratory applied calibrated QVA software capable of detecting diffuse, lower-grade disease extending beyond the segment the investigator judged to represent the target lesion, particularly given the substantial calcification and two chronic total occlusions present in this cohort. Both the dissection-grading and lesion-length discrepancies underscore the value of independent core-laboratory assessment in early-phase device studies and suggest that investigator-reported procedural metrics alone may not reliably characterize vessel injury or lesion complexity in similarly complex femoropopliteal disease.

### 4.7. Twelve-Month Safety, Patency, and Functional Outcomes

No deaths, major or minor target limb amputations, cd-TLRs, or cd-TVRs occurred throughout the 12 months, and freedom from the prespecified MAE composite was 100%. Thirteen adverse events occurred, including four serious adverse events in two participants. There were no serious adverse device effects, unanticipated serious adverse device effects, anticipated serious adverse device effects, or device deficiencies. The appropriate interpretation is that no new safety signal was identified, rather than that population-level safety has been conclusively established.

No binary restenosis was observed at 6 months. One core-laboratory-confirmed restenosis was identified during the 12-month visit window, resulting in an observed visit-based primary patency of 83.3%. The event occurred on day 391, so the Kaplan–Meier estimate at exactly day 365 remained 100%. This distinction should be preserved because the two estimates describe different analytical time frames rather than contradictory results. The absence of cd-TLR despite the duplex-defined restenosis also emphasizes the distinction between anatomical restenosis and clinically driven repeat intervention.

Patient-reported and clinical functional outcomes showed consistent numerical improvement. Five of six participants demonstrated improvement of at least one Rutherford category at 12 months. Overall WIQ score increased from 21.87 at baseline to 62.28 at 12 months, with improvements across pain, distance, walking speed, and stair-climbing domains. Resting ABI increased substantially at 1 month but the mean improvement was less evident at 6 and 12 months. Because these data derive from six participants, no inferential efficacy claim should be based on changes in Rutherford classification, ABI, or WIQ.

### 4.8. Strengths and Limitations

Several methodological strengths deserve emphasis. Pharmacokinetic sampling was intensive and prospectively defined. Concentrations were analyzed using validated analytical methods and non-compartmental pharmacokinetic analysis. Angiographic and duplex-ultrasound outcomes were assessed independently by a core laboratory. Potential clinical endpoint events were independently adjudicated. Follow-up completeness was 100% over 12 months.

The major limitation is the sample size. Six participants were intentionally enrolled for pharmacokinetic characterization, and this study was not statistically powered for safety or efficacy endpoints. With only six participants, point estimates such as 100% freedom from revascularization or 83.3% visit-based primary patency are intrinsically unstable—a single additional event would materially change each percentage—and must not be interpreted as evidence of clinical efficacy or long-term safety. BRight PK is best understood as a pharmacokinetic/feasibility study with descriptive, hypothesis-generating clinical follow-up, not as a clinical efficacy trial. This study was single-center, non-randomized, and lacked a contemporaneous comparator. Lesion characteristics were heterogeneous, with substantial variation in lesion length and calcification. Blood sampling ended at 24 h, before a terminal sirolimus elimination phase was reached. Human arterial tissue concentrations were not measured. Drug loss during introduction through the sheath and transit to the target lesion—recognized for other DCB technologies—was not separately quantified in this study; only the proportion of drug remaining on the balloon after withdrawal was assessed (Section 3.6). Finally, one event in six participants changes an observed binary outcome by 16.7 percentage points; therefore, primary patency, restenosis, and adverse event proportions should not be interpreted as precise population estimates.

### 4.9. Clinical Implications and Future Directions

The findings provide an early translational assessment of a novel limus-based DCB strategy. The combination of rapid systemic BIOtorcin™ decline, low systemic sirolimus concentrations, efficient release from the balloon, and prolonged tissue retention demonstrated preclinically provides a pharmacological rationale for continued clinical development.

The broader femoropopliteal DCB landscape continues to evolve alongside this early-phase evaluation of BIOtorcin™. For example, Sallustro et al. reported more favorable 24-month outcomes with a paclitaxel–dextran DCB than with plain balloon angioplasty in patients with femoropopliteal disease, including higher primary patency (88.2% vs. 71.5%), lower restenosis/reocclusion (9.4% vs. 25.3%), and a lower clinically driven target lesion revascularization rate (7.5% vs. 18.3%) [41]. Although the drug, excipient, and coating technology differ substantially from BIOtorcin™, these findings illustrate how alternative DCB formulations and drug-delivery strategies can influence longer-term femoropopliteal outcomes and provide a clinical benchmark for future, adequately powered BRight DCB studies. The present six-participant BRight PK cohort was not designed, and is not intended, to establish comparative efficacy against this or any other DCB platform.

Future studies should include larger multicenter populations with sufficiently powered clinical endpoints, broader lesion complexity, and longer follow-up. Extended pharmacokinetic sampling beyond 24 h would allow more complete characterization of sirolimus metabolism and elimination, and future studies incorporating direct human arterial tissue sampling or other validated local-concentration methods would allow confirmation of the prolonged tissue persistence currently supported only by preclinical porcine data. Comparative studies against established DCB technologies will ultimately be required to determine whether the pharmacological properties of BIOtorcin™ translate into durable advantages in patency, target lesion revascularization, functional outcomes, or safety.

## 5. Conclusions

BIOtorcin™ is a novel synthetic, highly lipophilic derivative of sirolimus developed for local arterial delivery. In the BRight PK study, BIOtorcin™ showed rapid systemic appearance after BRight DCB treatment followed by progressive decline in circulating parent-drug concentrations, while systemic sirolimus exposure remained low within the 24 h sampling interval. Device delivery was successful in all treated balloons, and no new device-related safety signal was identified through 12 months. Given the pharmacokinetic design, single-center setting, and sample size of six participants, the clinical findings should be considered exploratory and hypothesis-generating rather than evidence of clinical efficacy or long-term safety. Larger, adequately powered studies are required to establish long-term safety and clinical effectiveness.

## Figures and Tables

**Table 1:** Baseline demographic and clinical characteristics.

Characteristic	Value
Age, years	70.8 ± 7.39
Male sex	4 (66.7%)
BMI, kg/m^2^	26.13 ± 5.10
Coronary artery disease	3 (50.0%)
Cerebrovascular disease	1 (16.7%)
Hypertension	4 (66.7%)
Hyperlipidemia	3 (50.0%)
Diabetes mellitus	3 (50.0%)
Renal disease	1 (16.7%)
Current smoker	4 (66.7%)
Former smoker	2 (33.3%)
Previous peripheral intervention/surgery	2 (33.3%)
Rutherford category 2/3/4	1/3/2

Note: Values are presented as mean ± SD or *n* (%), unless otherwise indicated. Rutherford category is presented as the number of participants in categories 2, 3, and 4, respectively. BMI, body mass index; SD, standard deviation.

**Table 2:** Core-laboratory lesion and procedural characteristics.

Characteristic	Value
SFA involvement	6 (100%)
Popliteal extension	2 (33.3%)
Stenosis/occlusion	4/2
Lesion length, mm	140.85 ± 136.23
Reference vessel diameter, mm	5.527 ± 0.409
Baseline diameter stenosis, %	78.50 ± 23.39
PACSS grade 3–4	4 (66.7%)
BRight DCBs deployed	7
Mean DCB inflation time, s	207.4 ± 37.41
Post-dilatation	2 (33.3%)
Bailout stenting	0
Baseline MLD, mm	1.248 ± 1.368
Final MLD, mm	3.963 ± 0.594
Final residual stenosis, %	28.49 ± 7.64
Device success	7/7 (100%)
Acute technical success	4/6 (66.7%)
Acute procedural success	4/6 (66.7%)

Note: Values are presented as mean ± SD, *n* (%), or number of devices/lesions, as appropriate. Angiographic measurements were based on independent core-laboratory assessment. Device success was assessed per BRight DCB, whereas acute technical and procedural success were assessed per treated lesion/participant according to protocol definitions. DCB, drug-coated balloon; MLD, minimum lumen diameter; PACSS, Peripheral Arterial Calcium Scoring System; SFA, superficial femoral artery; SD, standard deviation.

**Table 3:** Pharmacokinetic parameters.

Characteristic	Value
BIOtorcin™ total exposure, µg	3976.8 ± 1665.7
BIOtorcin™ dose, mg/kg	0.051 ± 0.0188
BIOtorcin™ Cmax, ng/mL	23.60 ± 9.88
BIOtorcin™ Tmax, h	0.08
BIOtorcin™ AUC_0_–t, h·ng/mL	28.30 ± 12.19
BIOtorcin™ AUC_0_–∞, h·ng/mL	47.47 ± 18.27
BIOtorcin™ λz, h^−1^	0.0383 ± 0.0172
BIOtorcin™ t½, h	21.87 ± 10.17
Sirolimus Cmax, ng/mL	0.692 ± 0.462
Sirolimus Tmax, h	24
Sirolimus AUC_0_–t, h·ng/mL	9.307 ± 6.026
Sirolimus AUC_0_–∞/λz/t½	Not estimable

Note: Values are presented as mean ± SD unless otherwise indicated. Cmax and Tmax denote the maximum observed concentration and corresponding observed time point, respectively. BIOtorcin™ Cmax occurred at the first post-treatment sample (5 min; 0.08 h). Sirolimus concentrations were still increasing at the final 24 h sample; therefore, the reported sirolimus Cmax and Tmax are maxima observed within the sampling interval, and AUC0–∞, λz, and t½ could not be reliably estimated. AUC, area under the concentration–time curve; Cmax, maximum observed concentration; SD, standard deviation; Tmax, time to maximum observed concentration; λz, terminal elimination rate constant; t½, terminal elimination half-life.

**Table 4:** Twelve-month safety and patency outcomes.

Characteristic	Value
Overall survival	100%
Freedom from MAE	100%
Major target limb amputation	0
Minor target limb amputation	0
cd-TLR	0
cd-TVR	0
Binary restenosis at 6 months	0/6 (0%)
Binary restenosis at 12-month visit	1/6 (16.7%)
Visit-based 12-month primary patency	5/6 (83.3%)
Day-365 Kaplan–Meier primary patency	100%

Note: Values are presented as proportions or event counts, as appropriate. Primary patency was defined as duplex-ultrasound PSVR ≤2.5 or angiographic stenosis ≤50% together with freedom from cd-TLR. One binary restenosis was identified at day 391, within the protocol-defined 12-month visit window but after calendar day 365; therefore, visit-based 12-month primary patency was 83.3% (5/6), whereas the Kaplan–Meier estimate at day 365 was 100%. cd-TLR, clinically driven target-lesion revascularization; cd-TVR, clinically driven target-vessel revascularization; MAE, major adverse event; PSVR, peak systolic velocity ratio.

**Table 5:** Clinical and functional outcomes over 12 months.

Outcome	Baseline	1 Month	6 Months	12 Months
Rutherford category, mean change	—	−2.50 ± 0.837	−2.33 ± 1.211	−1.83 ± 1.941
ABI	0.723 ± 0.142	0.880 ± 0.135	0.734 ± 0.100 *	0.752 ± 0.205
WIQ overall	21.87 ± 19.16	48.53 ± 33.96	54.08 ± 31.81	62.28 ± 38.78

Note: Values are mean ± SD. Rutherford values represent mean paired change from baseline; negative values indicate improvement to a lower Rutherford clinical category. ABI and WIQ values represent absolute measurements at each visit. * ABI at 6 months was available for five participants. Higher WIQ scores indicate better self-reported walking function. ABI, ankle–brachial index; SD, standard deviation; WIQ, Walking Impairment Questionnaire.

## Data Availability

De-identified participant-level data may be made available upon reasonable request, subject to sponsor authorization and applicable ethics, governance, and privacy requirements.

## Supplementary Materials

The following supporting information can be downloaded at https://www.mdpi.com/article/10.3390/biomedicines14102359/s1: Table S1: Participant-level BIOtorcin™ exposure; Table S2: Rutherford clinical category distribution at baseline and 1, 6, and 12 months; Table S3: Detailed target limb ankle–brachial index values, clinical classification, and paired changes from baseline; Table S4: Detailed Walking Impairment Questionnaire domain scores; Table S5: Adverse event summary; Figure S1: BIOtorcin™ pharmacokinetic parameter summary; Figure S2: Rutherford clinical category distribution over time. TREND Statement Checklist [42].
