# Supplementary material for: Pharmacokinetics, Safety, and Exploratory 12-Month Clinical Follow-Up of a Novel BIOtorcin™-Coated Balloon for Femoropopliteal Artery Disease: The BRight PK Study
Source: Biomedicines. 2026 Oct 16;14(10):2359. doi: 10.3390/biomedicines14102359 (PMC13702093; doi:10.3390/biomedicines14102359)
Supplement: Supplementary file 1 [file biomedicines-14-02359-s001.zip › biomedicines-4613754-supplementary.pdf]

BRight PK Study — Supplementary Materials

Pharmacokinetics, Safety, and Exploratory 12-Month Clinical Follow-Up of a Novel BIOtorcin™-Coated Balloon for Femoropopliteal Artery Disease: The BRight PK Study

Manuscript ID: biomedicines-4613754

Table S1, Participant-level BIOtorcin™ exposure; Table S2, Rutherford clinical category distribution at baseline and 1, 6, and 12 months; Table S3, Detailed target limb ankle–brachial index values, clinical classification, and paired changes from baseline; Table S4, Detailed Walking Impairment Questionnaire domain scores; Table S5, Adverse event summary; Figure S1, BIOtorcin™ pharmacokinetic parameter summary; Figure S2, Rutherford clinical category distribution over time.

Table S1. Participant-level BIOtorcin™ exposure.

Author note: the per-participant BIOtorcin™ dose, exposure, and balloon-residual dataset is being finalized against the locked trial database and will be inserted here as a six-row table (one row per participant) immediately prior to final submission. The following group-level summary values are already reported in the main manuscript (Section 3.6; Table 3) and are reproduced here for reference pending the participant-level table: mean total BIOtorcin™ exposure 3976.8 ± 1665.7 µg (range 2761–7205 µg); mean body weight–normalized dose 0.051 ± 0.0188 mg/kg (range 0.037–0.088 mg/kg); five participants received a single BRight DCB and one participant received two 120 mm balloons (highest exposure, 7205 µg / 0.088 mg/kg). Participant-level analyses showed a low proportion of drug remaining on the balloon after deployment, consistent with substantial release from the device; drug loss during sheath introduction and arterial transit was not separately quantified (Section 3.6; Section 4.8).

Table S2. Rutherford clinical category distribution at baseline and 1, 6, and 12 months.

Rutherford category	Baseline, n	1 month, n	6 months, n	12 months, n
0	0	4	3	3
1	0	1	1	0
2	1	0	2	2
3	3	1	0	0
4	2	0	0	1
Total (N)	6	6	6	6

Values are participant counts (n) at each visit. Five of six participants had a lower Rutherford category at 12 months than at baseline. Mean paired change from baseline was −2.50 ± 0.837 at 1 month, −2.33 ± 1.211 at 6 months, and −1.83 ± 1.941 at 12 months (Section 3.12). Source: manuscript Section 3.12.

Table S3. Detailed target limb ankle–brachial index (ABI) values, clinical classification, and paired changes from baseline.

Measure	Baseline	1 month	6 months	12 months
N	6	6	5	6
Mean ± SD	0.723 ± 0.1418	0.880 ± 0.1349	0.734 ± 0.0999	0.752 ± 0.2047

Measure	Baseline	1 month	6 months	12 months
Min–max	0.58–0.97	0.71–1.02	0.60–0.86	0.42–0.97
95% CI	0.5745–0.8721	0.7384–1.0216	0.6100–0.8580	0.5369–0.9655
Severe (0–0.4), n (%)	0 (0.0%)	0 (0.0%)	0 (0.0%)	0 (0.0%)
Moderate (0.41–0.69), n (%)	3 (50.0%)	0 (0.0%)	1 (20.0%)	2 (33.3%)
Mild (0.7–0.89), n (%)	2 (33.3%)	3 (50.0%)	4 (80.0%)	2 (33.3%)
Normal (0.9–1.3), n (%)	1 (16.7%)	3 (50.0%)	0 (0.0%)	2 (33.3%)
Paired change vs. baseline, mean ± SD	—	+0.16 ± 0.135	+0.01 ± 0.116	+0.03 ± 0.194

ABI classification denominators reflect available measurements at each visit (6-moonth n = 5). The largest mean hemodynamic improvement was observed at 1 month and was not sustained to the same magnitude at later follow-up. Source: manuscript Section 3.13.

Table S4. Detailed Walking Impairment Questionnaire (WIQ) domain scores.

WIQ domain	Baseline, mean ± SD	12 months, mean ± SD
Pain	33.33 ± 25.82	62.50 ± 41.08
Walking distance	42.83 ± 29.63	71.50 ± 44.68
Walking speed	17.93 ± 18.60	55.62 ± 38.56
Stair-climbing	4.86 ± 10.01	59.72 ± 40.02

Overall WIQ score (all domains combined), by visit:

Visit	Overall WIQ, mean ± SD	Paired change vs. baseline, mean ± SD
Baseline	21.87 ± 19.16	—
1 month	48.53 ± 33.96	+26.66 ± 26.41
6 months	54.08 ± 31.81	+32.21 ± 37.63
12 months	62.28 ± 38.78	+40.40 ± 40.41

Higher scores indicate better self-reported walking function (0–100 scale). Domain-level scores are reported at baseline and 12 months as presented in the manuscript (Section 3.14); domain-level scores at 1 and 6 months were not separately reported in the main text. N = 6 at all visits. Source: manuscript Section 3.14; Table 5; Figure 4.

Table S5. Adverse event summary.

Category	Events, n	Participants affected, n
Total adverse events (all)	13	≤ 6
Serious adverse events, total	4	2
Gastrointestinal disorders (serious)	2	—
Other (including bone fracture)	2	—
Non-serious adverse events	9	—
Serious adverse device effects	0	0
Unanticipated serious adverse device effects	0	0

Category	Events, n	Participants affected, n
Anticipated serious adverse device effects	0	0
Device deficiencies	0	0

A total of 13 adverse events occurred among the six participants during 12-month follow-up; four were serious adverse events occurring in two participants (two gastrointestinal disorders, two other events including bone fracture). No pattern of serious toxicity attributable to BIOtorcin™ or sirolimus was identified. A full breakdown of the nine non-serious adverse events by system organ class was not reported in the main text and can be added from the clinical database if required by the editor. Source: manuscript Section 3.15.

Figure S1. BIOtorcin™ pharmacokinetic parameter summary.

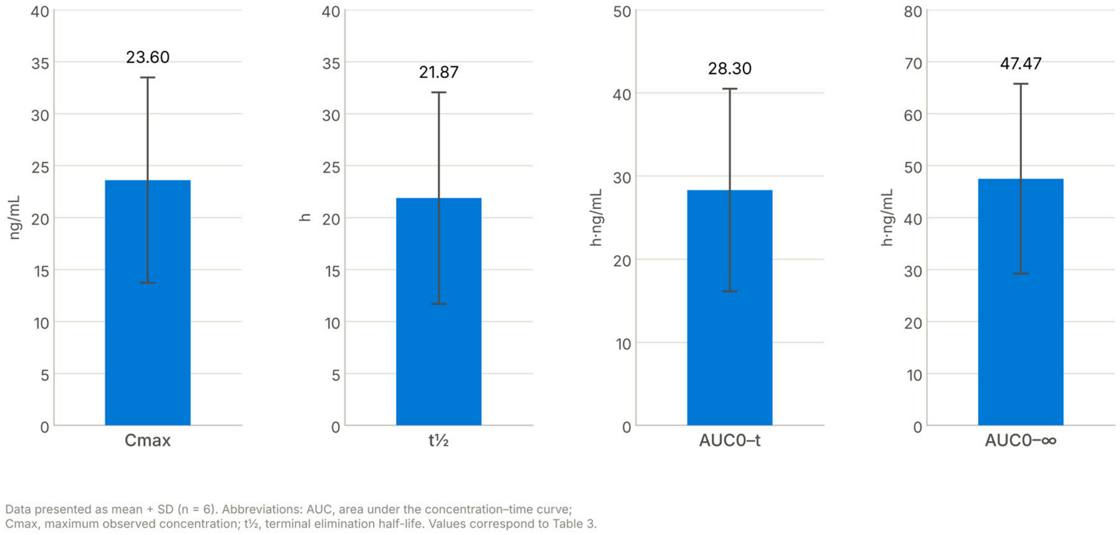

Figure S1. BIOtorcin™ pharmacokinetic parameter summary. Mean + SD BIOtorcin™ Cmax, terminal half-life (t½), AUC0–t, and AUC0–∞ following BRight DCB treatment (n = 6). Each panel uses its own axis and unit, as these parameters are not directly comparable on a shared scale. Values correspond to Table 3 of the main manuscript. Abbreviations: AUC, area under the concentration–time curve; Cmax, maximum observed concentration; SD, standard deviation; t½, terminal elimination half-life.

Figure S2. Rutherford clinical category distribution over time.

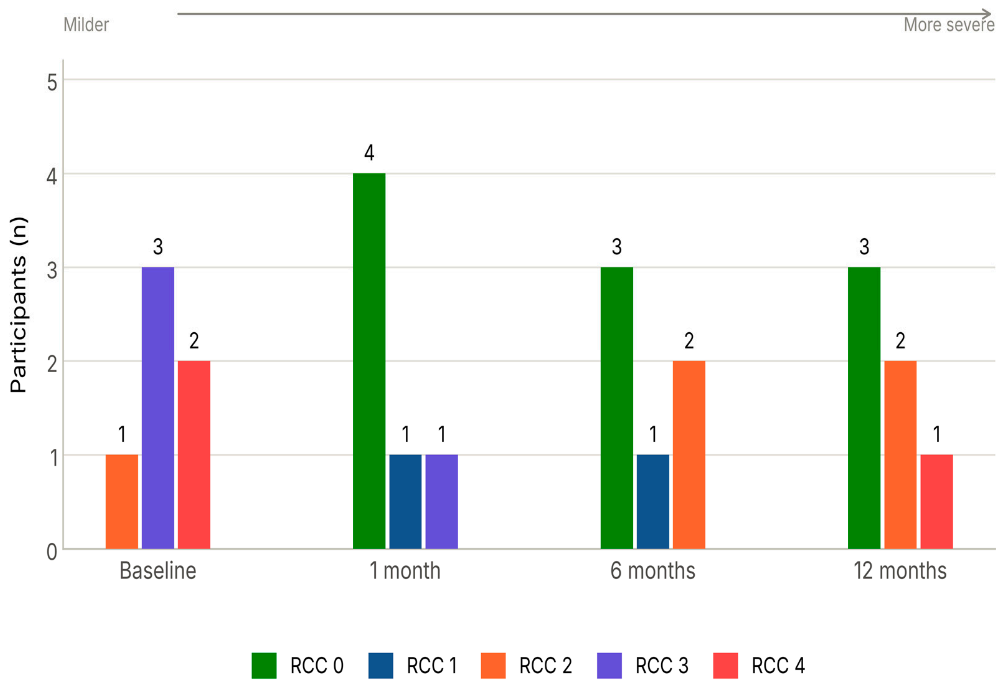

Figure S2. Rutherford clinical category (RCC) distribution over time. Number of participants in each Rutherford–Becker category at baseline and at 1, 6, and 12 months after BRight DCB treatment (n = 6 at each visit). Values correspond to Table S2 and manuscript Section 3.12.

TREND Statement Checklist

Transparent Reporting of Evaluations with Nonrandomized Designs

BRight PK Study: Pharmacokinetics, Safety, and 12-Month Clinical Performance of a Novel BIOtorcin™-Coated Balloon for Femoropopliteal Artery Disease

Study design: prospective, single-center, single-arm, open-label, non-randomized interventional pharmacokinetic clinical study.
ClinicalTrials.gov: NCT06065345. East Metropolitan Health Service Research Governance: RGS0000006363.

Paper section / topic	Item	TREND descriptor	Reported?	Manuscript location
Title and Abstract	1	Allocation method/design, target population or study sample, and structured abstract.	Yes	Title and Abstract
Introduction — Background	2	Scientific background, rationale, and theory/mechanism underlying the intervention.	Yes	Introduction; Figure 2
Methods — Participants	3	Eligibility criteria; recruitment method and setting; settings and locations of data collection.	Yes	Methods 2.1–2.2; Figure 1
Methods — Interventions	4	Intervention content, delivery method, unit/deliverer/setting, exposure quantity and duration, time span, and measures supporting protocol adherence.	Yes	Methods 2.3–2.4; Figure 1; Table 2
Methods — Objectives	5	Specific objectives and hypotheses.	Yes	End of Introduction; Methods 2.8
Methods — Outcomes	6	Clearly defined primary and secondary outcomes; data-collection methods and measurement quality/validated instruments.	Yes	Methods 2.5–2.7
Methods — Sample Size	7	How sample size was determined; interim analyses/stopping rules where applicable.	Yes	Methods 2.8 (feasibility/PK sample; no interim analysis)
Methods — Assignment Method	8	Unit and method of assignment to study condition; restrictions or methods used to limit bias from non-randomization.	N/A / Yes	Methods 2.1: single-arm study; no random allocation or comparator
Methods — Blinding (Masking)	9	Whether participants, intervention providers, and outcome assessors were blinded; method of blinding if used.	Yes	Methods 2.1: open-label; independent core laboratory/CEC assessment
Methods — Unit of Analysis	10	Smallest unit analyzed and methods used if unit of analysis differs from unit of assignment.	Yes	Methods 2.8; Tables 2–5
Methods — Statistical Methods	11	Statistical methods for primary/additional analyses, missing data, and statistical software.	Yes	Methods 2.6 and 2.8
Results — Participant Flow	12	Flow through enrollment, intervention exposure, follow-up, and analysis; protocol deviations; flow diagram recommended.	Yes	Results 3.1; Figure 1
Results — Recruitment	13	Dates defining recruitment and follow-up periods.	Yes	Methods 2.1; Results 3.1
Results — Baseline Data	14	Baseline demographic/clinical characteristics and relevant comparisons.	Yes	Results 3.2–3.4; Tables 1–2
Results — Baseline Equivalence	15	Study-group equivalence at baseline and methods used to control baseline differences.	N/A	Single-arm study; no comparator group
Results — Numbers Analyzed	16	Denominators for each analysis; absolute numbers; analysis strategy and handling of non-compliers.	Yes	Results 3.1 and outcome subsections; Tables 1–5
Results — Outcomes and Estimation	17	Primary and secondary outcome results, precision where appropriate, null/negative findings, and prespecified causal pathways if tested.	Yes	Results 3.6–3.15; Tables 3–5; Figures 3–5
Results — Ancillary Analyses	18	Other analyses, including subgroup/restricted analyses, identified as prespecified or exploratory.	Yes / N/A	Methods 2.8; exploratory/descriptive clinical outcomes; no subgroup modelling
Results — Adverse Events	19	Important adverse events and unintended effects.	Yes	Results 3.15; Supplementary Table S5
Discussion — Interpretation	20	Interpretation considering hypotheses, bias, imprecision, multiplicity, limitations, mechanism, implementation, and implications.	Yes	Discussion 4.1–4.8; Conclusions
Discussion — Generalizability	21	External validity considering population, intervention, follow-up, setting, and contextual factors.	Yes	Discussion 4.8
Discussion — Overall Evidence	22	Overall interpretation in the context of current evidence and theory.	Yes	Discussion 4.2–4.7; Conclusions

Notes specific to this study: The BRight PK Study had one treatment group only; therefore, TREND items concerning between-group assignment or baseline equivalence are not applicable. Clinical outcomes were secondary/exploratory and are reported descriptively because the study was designed primarily for pharmacokinetic characterization.

Source: Des Jarlais DC, Lyles C, Crepaz N, and the TREND Group. TREND Statement Checklist (2004). Checklist completed for the BRight PK Study.
